# Effects of Glutamine Dipeptide-Supplemented Formulated Diet Substituting Chilled Trash Fish in Chinese Mitten Crab (*Eriocheir sinensis*)

**DOI:** 10.3390/biology15010080

**Published:** 2025-12-31

**Authors:** Wenjun Qiu, Xueming Hua, Bin Luo, Huanchao Ma, Ying Hang, Saiya Liu, Dong Yu, Shuichao Mi, Jun Zhang, Jie Yang, Jianbin Zu

**Affiliations:** 1 Centre for Research on Environmental Ecology and Fish Nutrion of the Ministry of Agriculture and Rural Affairs, Shanghai Ocean University, Shanghai 201306, China; 15618152389@163.com (W.Q.); m230100303@st.shou.edu.cn (B.L.); m220100321@st.shou.edu.cn (H.M.); m230150424@st.shou.edu.cn (S.L.); m230100204@st.shou.edu.cn (D.Y.); 2 Key Laboratory of Freshwater Aquatic Genetic Resources, Ministry of Agriculture and Rural Affairs, Shanghai Ocean University, Shanghai 201306, China; 3 National Demonstration Center for Experimental Fisheries Science Education, Shanghai Ocean University, Shanghai 201306, China; 4 Changzhou Yayuan Biochemical Technology Co., Ltd., Changzhou 213115, China; shmdjj@126.com (S.M.); dylan_zhang@163.com (J.Z.); 5 Shanghai Chongming Fisheries Technical Extension Station, Shanghai 202150, China; jieyang1880822@163.com (J.Y.); cmxdny@163.com (J.Z.)

**Keywords:** *Eriocheir sinensis*, glutamine dipeptide, growth, compound feed, fresh-frozen fish, health, edible quality

## Abstract

Feeding Chinese mitten crabs with fresh-frozen fish causes nutritional imbalance and increases disease risk. Compound feed offers better nutrient balance but still requires improvements in palatability and growth performance. This study evaluated the effects of replacing fresh-frozen fish with glutamine dipeptide-supplemented formulated diet on growth, hepatopancreas health, and edible quality, aiming to inform feed formulation strategies. A five-month feeding trial (June–October) was conducted with two treatments: the experimental group received only glutamine dipeptide compound feed, while the control group was fed a mix of fresh-frozen fish and compound feed. Crabs in the experimental group showed significantly higher body weight, length, and width. No significant differences were found in the hepatopancreatic index, gonadosomatic index, meat yield, or total edible yield. In October, the experimental group showed lower malondialdehyde (MDA) levels in the hepatopancreas and higher alkaline phosphatase (AKP) and acid phosphatase (ACP) activities in males. In females, hemolymph AKP and ACP were higher in the control, while glutamic pyruvic transaminase (GPT) was higher in the experimental group. Whether this is related to a potential risk of liver damage or a reaction at a special stage remains to be further verified. Digestive enzyme activities (protease, lipase, amylase) were generally higher in the experimental group, particularly in August (*p* < 0.05). In October, protease activity in females and lipase activity in males were significantly higher than in controls (*p* < 0.05). Nitrogen and phosphorus retention in muscle was also significantly higher, indicating better nutrient utilization (*p* < 0.05). Overall, these findings indicate that a glutamine dipeptide-supplemented diet provides a more effective and sustainable alternative to fresh-frozen fish over a five-month rearing period, improving digestive physiology, feed efficiency, growth performance, and edible quality and flavor.

## Simple Summary

This study evaluated a glutamine dipeptide-supplemented formulated diet as a sustainable alternative to fresh-frozen fish in Chinese mitten crab aquaculture. A five-month trial demonstrated improved growth performance, nutrient retention, and hepatopancreatic health, along with enhanced muscle quality characterized by increased umami- and sweet-tasting amino acids. The findings support the commercial viability of this dietary strategy, offering a sustainable solution to reduce reliance on wild-caught fish while improving production efficiency and product quality in crab aquaculture.

## 1. Introduction

The Chinese mitten crab (*Eriocheir sinensis*), commonly known as the river crab, is widely distributed throughout the Yangtze River and Yellow River basins, as well as eastern coastal watersheds in China. Valued by consumers for its distinctive umami–sweet flavor, it ranks among the nation’s most lucrative cultured crustaceans. National harvest reached 815,318 t in 2023 [1], and ongoing advances in husbandry continue to expand production. Despite these gains, reliance on wild “trash-fish” bait remains common. In 2023, China set a goal that ≥80% of baitfish be replaced by formulated diets in crab farming, yet farmer uptake has been slow. Many commercial feeds are designed to meet only minimal nutrient specifications at the lowest cost, resulting in batch-to-batch variation in ingredient profiles and inconsistent performance. Consequently, numerous producers still regard wild baitfish as more reliable [2]. Globally, about 5–6 million tonnes of wild fish are directly used to feed farmed fish and crustaceans, especially in China [3]. In Thailand, junk fish is also chosen as the main source of protein for fattening crabs and lobsters. Similar phenomena also exist in Vietnam and Cambodia [4,5]. Feeding fresh or frozen fish generates high organic loads, elevates ammonia-nitrogen and reactive phosphorus concentrations, and facilitates pathogen reproduction, ultimately degrading habitat quality and market traits such as size uniformity and shell coloration. Conversely, balanced compound diets typically yield lower feed-conversion ratios (FCRs) and reduce effluent nutrients [6]. In Chinese mitten crab aquaculture, conventional fresh fish feeding demonstrates feed-conversion ratios (FCRs) of 2.5–4.0 [7], while sustainable pellet formulations achieve superior FCRs (<1.3) alongside enhanced nitrogen and phosphorus retention efficiencies [8]. The hybrid feeding protocol, combining pellet feeds in nursery phases with controlled fresh fish supplementation during grow-out, significantly improves male gonadosomatic indices and female muscle textural properties relative to exclusive pellet feeding [9]. Therefore, the pellet feed currently used throughout the entire feeding process still needs to be optimized.

Formulating feeds with functional additives is a promising route to further improve performance [10]. Glutamine is a key energy substrate for intestinal mucosa [11], supplemental glutamine (0.4–1.6%) has enhanced growth and feed efficiency in loach [12], hybrid tilapia (*Oreochromis*) [13] and juvenile groupers (*Epinephelus marginatus*) [14]. However, glutamine has an antagonistic effect on the mTOR pathway in C_2_C_12_ myogenic cells, which may limit protein synthesis in the body and be unfavorable for growth [15]. The dipeptide alanyl-glutamine (Ala-Gln) is more stable and water-soluble than free glutamine [16], supports mucosal repair and immunity, and mitigates stress-related growth depression [17]. Dietary inclusion of 0.5–1.0% Ala-Gln has improved growth metrics in juvenile taimen (*Hucho taimen*) [18], mirror carp (*Cyprinus carpio* L.) [19], and whiteleg shrimp (*Penaeus vannamei*) [20]. However, some studies have pointed out that adding 1% and 2% glutamine dipeptide to the diet of *Macrobrachium rosenbergii* has no significant effect on its growth performance and MDA content [21]. Collectively, these findings may suggest that a glutamine dipeptide-fortified diet could both sustain growth and enhance health in crustaceans, depending on the amount of glutamine dipeptide added and species. In addition, some studies have found that under the same feeding method, the number of muscle fingers and fattening degree of male Chinese mitten crabs are higher than those of female crabs, while the number of liver and pancreatic gland fingers and the number of sex gland fingers are lower than those of female crabs [22]. Some studies have also shown that sexually mature male and female crabs have significantly different sex regulation patterns within their gonads [23]. Therefore, this study evaluated whether glutamine dipeptide compound feed could completely replace fresh baitfish in the farming of Chinese mitten crabs based on the differentiation of their gender, providing new ideas for the replacement of fresh baitfish in the global crustacean breeding process and achieving sustainable development. Two feeding regimes were tested: (i) a mixed ration of pellets and fresh fish, and (ii) an all-pellet diet containing the optimal glutamine dipeptide level determined in preliminary trials. Growth performance, nitrogen and phosphorus retention, hepatopancreas and gut histology, and edible tissue quality (muscle, hepatopancreas, gonad) were compared to assess the feasibility of complete baitfish substitution throughout the production cycle.

## 2. Materials and Methods

### 2.1. Experimental Feed and Aquaculture Management

The control group was fed a mixed diet of fresh fish and compound feed throughout the experiment. Both fresh fish and compound feed were provided by the experimental base. The experimental group was fed exclusively with compound feed containing 2% glutamine dipeptide, entrusted to Nanjing Foster Feed Company for processing. This glutamine dipeptide was co-developed by Changzhou Yayuan Biotechnology Co., Ltd. and Shanghai Ocean University and was processed into a compound feed by a feed production company. The moisture, ash, crude protein, and crude fat contents (%) of the feed in the control group, respectively, were 9.72 ± 0.06; 9.28 ± 0.05; 45.58 ± 0.07; and 2.84 ± 0.55; those for the experimental group were 11.40 ± 0.74; 10.64 ± 0.03; 42.09 ± 0.13; and 6.43 ± 0.58.

The feeding trial was conducted from early June to late October 2024 at Chongming Huikang Aquatic Farming Cooperative, Shanghai, China. Juvenile Chinese mitten crabs at the second post-molt stage were used in the trial. Initial body mass averaged 77.2 ± 9.5 g for males and 74.7 ± 6.7 g for females. The crabs were then randomly distributed into two earthen ponds: 3300 m^2^ for the conventional-diet group and 6000 m^2^ for the glutamine dipeptide group. Animals were hand-fed once daily at 16:00 h for the entire five-month period, and dissolved oxygen was maintained above 5 mg L^−1^ by mechanical aeration. Due to the limited experimental conditions available at the research site, it was not feasible to use ponds of uniform size, and therefore, this study did not include replicated experiments. To mitigate the potential confounding effects arising from variations in pond size and to enhance the reliability of the experimental results under these constraints, the following measures were implemented: the two experimental ponds were situated adjacent to each other, and the initial stocking density (approximately 1.50 individuals/m^2^) of the experimental animals was kept identical in both ponds.

### 2.2. Experimental Indicators and Methods

#### 2.2.1. Sample Collection

During the farming period, monthly water quality tests were conducted. Crabs were sampled in August and at the end of the trial (October). For each treatment, 15 males and 15 females were netted at random. Individual crab weights were measured using an electronic balance, and body length and width were measured with calipers. Hemolymph was extracted from the base of the third pereopod using a sterile syringe. Live dissections were performed to collect hepatopancreas, gonads, and muscle samples, which were then weighed to calculate various growth metrics. All samples were stored at −20 °C.

#### 2.2.2. Growth Performance Assessment

At the end of the experiment (October), gonadosomatic index (GSI), muscle yield (MY), hepatopancreatic index (HSI), and total edible yield (TEY) were calculated:

(1) $$\mathrm{GSI}(\%)\;=\;W_{g}/W\times 100$$

(2) $$\mathrm{MY}(\%)=W_{m}/W\times 100$$

(3) $$\mathrm{HSI}(\%)=W_{h}/W\times 100$$

TEY(%) = GSI + MY + HSI (4)

Notes: W represents individual crab weight (g); $W_{g}$ represents the weight of the crab’s gonad (g); $W_{m}$ represents the weight of the crab’s muscle; $W_{h}$ represents the weight of the crab’s hepatopancreas (g).

For NRR (nitrogen retention rate) and PRR (phosphorus retention rate):

(5) NRR = [Wt×Dt×Nt−W0×D0×N0]/F×Df×Nf×100^%

(6) $$\mathrm{PRR}=[W_{t}\times D_{t}\times P_{t}-W_{0}\times D_{0}\times P_{0}]/F\times D_{f}\times P_{f}\times 100\%$$

Notes: $W_{t},D_{t}{,N}_{t},P_{t},$ represent the final body weight (g), dry matter content (%), and nitrogen/phosphorus concentrations (mg/g) in muscle tissue at the end of the experiment. $W_{0},D_{0},N_{0},P_{0},$ denote the initial body weight (g), dry matter content (%), and nitrogen/phosphorus concentrations (mg/g) in muscle tissue at the beginning of rearing. $F,D_{f},N_{f},P_{f},$ indicate the total feed consumption, not adjusted for the amount ingested by non-experimental animals in the ponds (g), dry matter content (%), and nitrogen/phosphorus concentrations (mg/g) in feed during the experimental period.

#### 2.2.3. Hepatopancreatic and Intestinal Health

Activities of hepatopancreatic acid phosphatase (ACP), alkaline phosphatase (AKP), glutamic pyruvic transaminase (GPT), and glutamic oxaloacetic transaminase (GOT), together with malondialdehyde (MDA) content, were assayed in hemolymph and hepatopancreas using commercial kits produced by the Nanjing Jiancheng Bioengineering Institute. Protease, amylase, and lipase activities in the hepatopancreas were also determined using commercial kits produced by the same supplier, Nanjing Jiancheng Institute of Bioengineering. Their specific numbers are as follows: ACP(A060-1-1), AKP (A059-1-1), GPT (C009-2-1), GOT (C010-2-1), MDA (A003-1-1), protease (A080-2-2), amylase (C016-1-2), and lipase (A054-1-1). For histology, tissues were fixed in Bouin’s solution for 24 h, dehydrated through graded ethanol, embedded in paraffin, sectioned at 5 µm, and stained with hematoxylin–eosin (H&E) [24]. Then the sections were observed using an ECLIPSE brand Ci-L microscope with a magnification of 200 times.

#### 2.2.4. Quality of Edible Parts (Muscle, Hepatopancreas, Gonad)

Proximate composition (moisture, crude protein, lipid, ash) and free amino acids in the muscles, hepatopancreas, and gonad were determined according to AOAC standard [25,26,27,28,29] nos. 950.46, 981.10, 963.15, and 982.30, respectively. For taste analysis, 5 g of each sample was homogenized in 30 mL ultrapure water, subjected to ultrasonic treatment for 4 min, left to stand for 30 min, and centrifuged (15,000 r/min, 15 min, 4 °C). The supernatant was filtered, adjusted to a volume of 100 mL, and analyzed using an electronic tongue (ASTREE system, Alpha M.O.S, Toulouse, France).

### 2.3. Data Analysis

All data were statistically analyzed using SPSS 24.0 (IBM, Armonk, NY, USA). After testing for normality and homogeneity of variance, data were analyzed by the independent samples t-test. For data that did not meet normality or homogeneity of variance assumptions, the Mann–Whitney U test was used. The confidence interval was set at 95%, and statistical significance was defined as *p* < 0.05. Results are presented as means ± standard deviation (Means ± SD).

## 3. Results and Analysis

### 3.1. Growth Performance Under Two Feeding Regimes

As shown in Table 1 and Table 2, in August, the body weight, length, and width of male and female crabs in the experimental group were significantly higher than those in the control group (*p* < 0.05). In October, the body weight of both male and female crabs in the experimental group was significantly greater than that of the control group (*p* < 0.05). Additionally, the body length and width of female crabs in the experimental group were also significantly higher (*p* < 0.05). No significant differences were observed in the hepatopancreas index, gonadosomatic index, meat yield, or total edible yield (*p* > 0.05).

**Figure 1:**
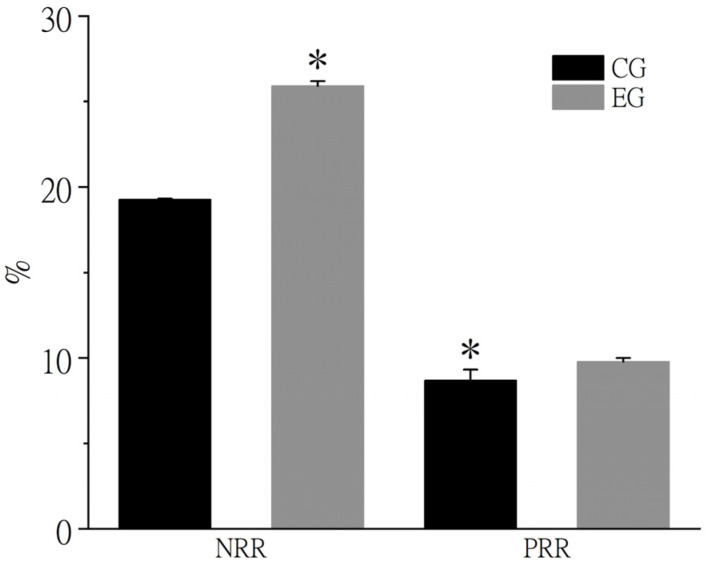
Retention rate of nitrogen and phosphorus in muscle of Chinese mitten crab in October. Note: * above the column indicates a significant difference between the control group and the experimental group (*p* < 0.05). CG means control group, EG means experimental group. NRR means nitrogen retention rate, PRR means phosphorus retention rate.

As shown in Figure 1, the nitrogen retention rate (NRR) in crab muscle significantly increased from 19.29% in the control group to 25.90% in the experimental group in October, while the phosphorus retention rate (PRR) rose significantly from 8.69% to 9.78% (*p* < 0.05).

### 3.2. Hepatopancreas and Intestinal Health of Chinese Mitten Crab Under Two Feeding Regimes

#### 3.2.1. Hepatopancreatic Metabolic Capacity

As shown in Table 3, in October, male crabs in the experimental group exhibited significantly higher hepatopancreatic alkaline and acid phosphatase activities compared to those in the control group (*p* < 0.05). In both August and October, male crabs in the control group showed significantly higher malondialdehyde (MDA) content in the hepatopancreas compared to the experimental group (*p* < 0.05). For female crabs, hepatopancreatic MDA content was significantly higher in the experimental group in August but reversed in October compared to the control group (*p* < 0.05). Additionally, in August, male crabs in the experimental group exhibited significantly higher hepatopancreatic alanine aminotransferase (GPT) activity compared to the control group (*p* < 0.05). In October, female crabs in the experimental group showed significantly elevated hepatopancreatic GPT activity relative to the control group (*p* < 0.05). No significant differences were detected in other hepatopancreatic enzyme activities between groups (*p* > 0.05).

#### 3.2.2. Hemolymph Metabolism

As shown in Table 4, male crabs in the experimental group exhibited significantly higher hemolymph alkaline phosphatase (ALP) and acid phosphatase (ACP) activities than the control group in October (*p* < 0.05). In October, female crabs in the experimental group showed reduced hemolymph malondialdehyde (MDA) levels compared to controls. During August, hemolymph alanine aminotransferase (GPT) activity in female crabs was significantly suppressed in the experimental group versus controls (*p* < 0.05). Furthermore, August data revealed elevated hemolymph ALP but diminished GPT activities in experimental female crabs relative to controls (*p* < 0.05). October data demonstrated significantly higher hemolymph ALP and ACP activities in control females versus the experimental group, whereas GPT activity showed an inverse pattern (*p* < 0.05).

#### 3.2.3. Histological Observation of Hepatopancreas

**Figure 2:**
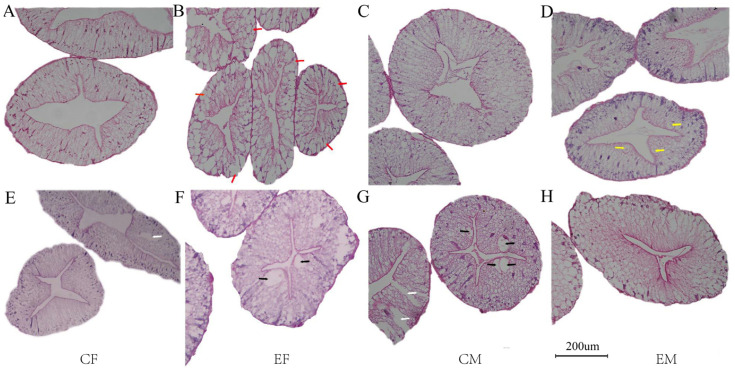
Hepatopancreas of Chinese mitten crab (*Eriocheir sinensis*). Note: The above scales are the same magnification of 200 times. (**A**–**D**) are samples from August; (**E**–**H**) are samples from October; red arrows show the absorptive cells; yellow arrows show bulliform cells; black arrows indicate cavitation; white arrows indicate that the cell has broken down and the cell line has disappeared.

In Chinese mitten crab, the hepatopancreas primarily comprises hepatic tubules with a simple single-layer columnar epithelium. A healthy hepatopancreas typically exhibits smooth, intact epithelium and connective tissue, complete basement membrane, and characteristic star-shaped lumen. In August, female crabs from the experimental group displayed numerous absorptive cells surrounding the basement membrane (Figure 2B). Located near the basement membrane at hepatic tubule bases, absorptive cells facilitate hemolymph nutrient absorption. August observations revealed bubble-like cells with transparent vacuoles adjacent to the hepatopancreatic lumen in male experimental crabs. These cells secrete abundant digestive enzyme precursors, with their luminal proximity promoting efficient release. In October, vacuolization occurred in both experimental females (Figure 2F) and control males (Figure 2G). October control groups exhibited cellular rupture and boundary loss in both sexes.

#### 3.2.4. Histological Observation of Intestines

**Figure 3:**
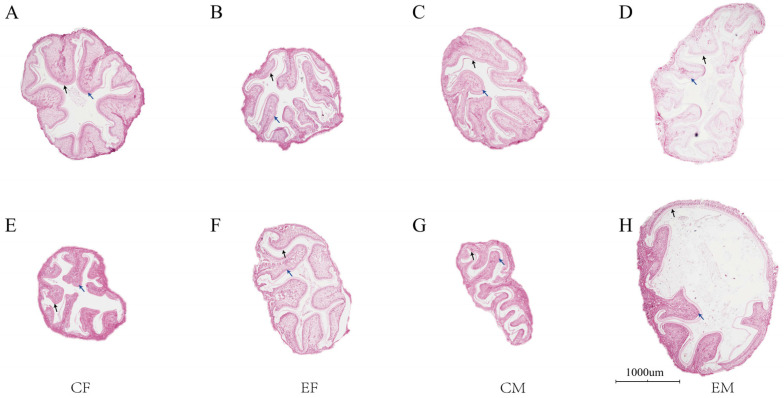
Intestinal tissue sections of Chinese mitten crab (*Eriocheir sinensis*). Note: The above scales are the same magnification of 200 times, (**A**–**D**) are samples from August; (**E**–**H**) are samples from October; black arrows indicate the peritrophic membrane; blue arrows show epithelial cells.

Plate II clearly demonstrates the food-bolus membrane and epithelial cells across groups, with consistently thin intestinal muscular layers in all specimens. Female crabs from the August control group (Figure 3A) and male crabs from the August experimental group (Figure 3D) exhibited partial detachment of the food-bolus membrane and epithelial cells in localized intestinal regions. Comparative analysis revealed superior intestinal health in the experimental group relative to controls.

#### 3.2.5. Hepatopancreatic Digestive Enzyme Activity

As shown in Table 5, in August, crabs in the experimental group exhibited significantly higher hepatopancreatic protease and lipase activities compared to the control group (*p* < 0.05). In October, male crabs in the control group showed significantly higher hepatopancreatic protease activity than those in the experimental group (*p* < 0.05), whereas amylase activity was significantly lower than that in the experimental group (*p* < 0.05). For female crabs, hepatopancreatic protease activity was significantly higher in the experimental group compared to the control group (*p* < 0.05), while lipase and amylase activities were significantly greater in the control group (*p* < 0.05).

### 3.3. Quality Evaluation of Edible Parts (Muscle, Hepatopancreas, and Gonads) Under Two Feeding Regimes

#### 3.3.1. Muscle Composition and Free Amino Acids

As shown in Table 6, the basic muscle composition of crabs under both feeding regimes did not differ significantly between groups, except for moisture content, which was significantly higher in female crabs of the experimental group compared to the control group.

Table 7 summarizes the concentrations of 16 flavor-associated free amino acids (FAAs) in crab muscle, revealing distinct differences between groups. Arginine (Arg, approximately 29 mg/g) and glycine (Gly, approximately 20 mg/g) were the most abundant, both classified as sweet-tasting.

In males, the control group had significantly higher levels of aspartic acid (Asp), methionine (Met), and proline (Pro), whereas the experimental group showed elevated levels of 11 FAAs, including Ser, His, Gly, Thr, Ala, Tyr, Val, Phe, Ile, Leu, and Lys (*p* < 0.05). In females, Tyr, Met, Leu, and Lys were higher in the control group, while Thr was elevated in the experimental group (*p* < 0.05). Other FAAs showed no significant differences (*p* > 0.05).

Although umami amino acid proportions were comparable between groups (*p* > 0.05), sweet-tasting amino acid content was significantly lower in experimental females (*p* < 0.05), suggesting a potential shift in flavor profile associated with dietary treatment.

#### 3.3.2. Free Amino Acids in the Hepatopancreas

As shown in Table 8, sixteen taste-related free amino acids were detected in the hepatopancreas, with arginine (Arg) and alanine (Ala) showing the highest concentrations (approximately 3 mg/g), both classified as sweet-tasting. In males, free amino acid levels did not differ significantly between groups (*p* > 0.05). In females, except for Ala, all amino acid concentrations were significantly lower in the experimental group (*p* < 0.05). Notably, the proportion of sweet-tasting amino acids was significantly higher in experimental males (*p* < 0.05), while both sweet and umami amino acid proportions were elevated in experimental females (*p* < 0.05).

#### 3.3.3. Free Amino Acids in the Gonads

As shown in Table 9, fifteen taste-related amino acids were identified in the gonads, with minor sex-specific differences: serine (Ser) was absent in males, and threonine (Thr) in females. Alanine (Ala, approximately 6.5 mg/g) and arginine (Arg, approximately 4.5 mg/g) were the most abundant in males and females, respectively.

In males, the experimental group showed significantly higher levels of glycine (Gly), tyrosine (Tyr), valine (Val), phenylalanine (Phe), isoleucine (Ile), leucine (Leu), and lysine (Lys), while methionine (Met) was higher in the control group (*p* < 0.05). In females, Ala and Tyr were significantly elevated in the experimental group, whereas Arg, Val, Met, Phe, Ile, Leu, and Lys were higher in controls (*p* < 0.05).

No significant difference was observed in umami amino acid proportions between groups (*p* > 0.05). However, total amino acid content was significantly higher in experimental males and lower in experimental females (*p* < 0.05). The proportion of sweet amino acids decreased in experimental males but increased in experimental females (*p* < 0.05).

#### 3.3.4. Taste Quality Evaluation of Muscle, Hepatopancreas, and Gonads Using an Electronic Tongue

**Figure 4:**
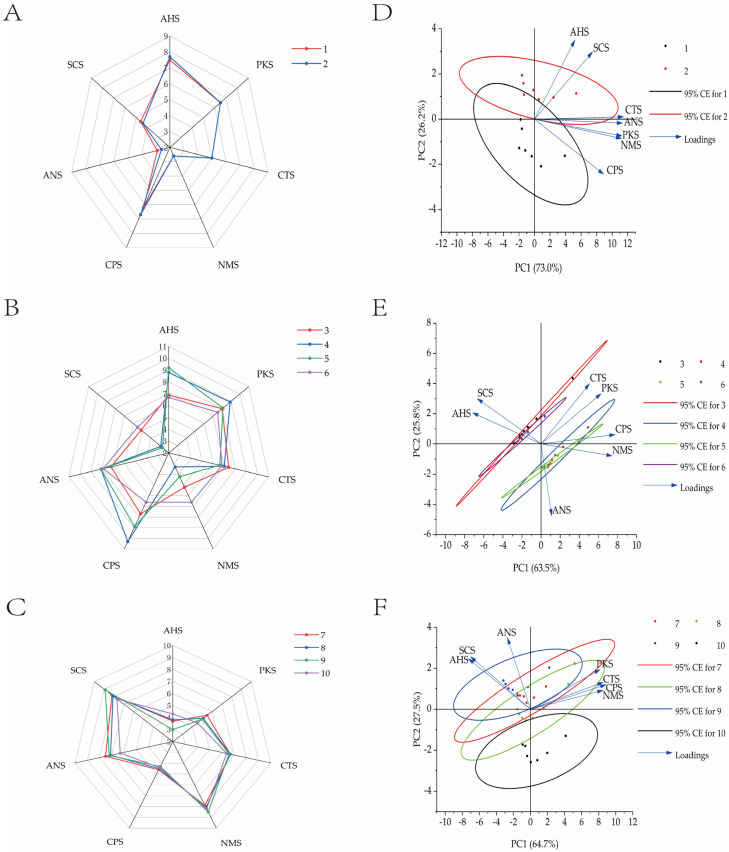
Taste quality of Chinese mitten crab gonads (**A**), hepatopancreas (**B**), and muscles (**C**). Taste PCA analysis diagram of Chinese mitten crab gonads (**D**), hepatopancreas (**E**), and muscles (**F**). Note: The right side of the figure is the PCA analysis diagram corresponding to the left sample; Nos. 1–10 represent, in sequence: (1) gonads of female crabs in control group; (2) gonads of female crabs in experimental group; (3) hepatopancreas of female crabs in control group; (4) hepatopancreas of female crabs in experimental group; (5) hepatopancreas of male crabs in control group; (6) hepatopancreas of male crabs in experimental group; (7) muscles of female crabs in control group; (8) muscle of female crabs in experimental group; (9) muscle of male crabs in control group; (10) muscle of male crabs in experimental group.

To further evaluate whether the taste quality of edible tissues differed significantly between groups, PCA was conducted to examine the spatial distribution of taste signals. The cumulative variance contribution exceeded 85%, indicating that Figure 4D–F sufficiently captured the key taste information of the samples.

In Figure 4D, PCA of female crab gonads revealed both similarities and differences between the experimental and control groups. Along PC1, the two groups displayed considerable overlap, while along PC2, distinct separation was observed: control group samples clustered on the positive side, while experimental group samples clustered on the negative side. PC2 was positively correlated with AHS (sour) and SCS (sweet) and negatively with CPS (bitter), suggesting that the control group exhibited stronger sour and sweet attributes, while bitterness was more pronounced in the experimental group. However, these differences were not statistically significant.

In Figure 4E, the hepatopancreas taste profiles were largely consistent between feeding regimes but significantly influenced by sex. Female crab samples primarily clustered on the negative side of PC1 and the positive side of PC2, indicating dominant contributions from SCS (sweet) and AHS (sour), and a lesser influence from ANS (astringent). In contrast, male samples showed stronger PKS (pungent), CTS (salty), CPS (bitter), and NMS (umami) responses, with ANS (astringent) more evenly distributed.

In Figure 4F, muscle taste profiles showed strong consistency within the same gender across feeding regimes, although specific taste differences were noted. In female crabs, the experimental group demonstrated elevated PKS (pungent), CTS (salty), CPS (bitter), and NMS (umami) responses, while the control group exhibited higher ANS (astringent), SCS (sweet), and AHS (sour) responses. In male crabs, the experimental group showed stronger ANS, SCS, and AHS responses, whereas the control group presented a more balanced distribution across sensors.

## 4. Discussion

### 4.1. Effects of Two Feeding Regimens on Growth Performance of Chinese Mitten Crab

In the present study, glutamine dipeptide supplementation significantly enhanced growth performance, particularly in females. This indicates that the modified diet supported more effective somatic development.

Glutamine, as a conditionally essential amino acid, may not be able to meet the demand when its own synthesis rate is insufficient under adverse conditions. Dietary nutrition critically influences the growth and economic viability of Chinese mitten crab aquaculture [30,31]. Therefore, supplemental glutamine dipeptide is rapidly hydrolyzed to glutamine in vivo [32], which enhances intestinal amino acid absorption and supports systemic protein synthesis [33]. This process may rectify amino-acid imbalances that otherwise hinder protein turnover and growth [34,35]. The elevated nitrogen retention observed in the supplemented group further underscores the diet’s capacity to improve overall nitrogen utilization. Given the stringent essential amino acid demands of crustaceans [36], glutamine dipeptide inclusion likely corrected dietary imbalances and enhanced intestinal uptake, thereby supporting normal physiological processes and somatic growth. The resulting enhancement in nitrogen conversion should also reduce the amount of nitrogenous waste entering the culture water, indicating that glutamine dipeptide-fortified formulated feed can serve as a practical substitute for frozen fish in intensive *E. sinensis* culture.

### 4.2. Effects of Feeding Regimens on Hepatopancreatic Digestive Enzymes

In August, the hepatopancreatic activities of protease and lipase were significantly higher in the experimental group than in the control group and the amylase was also higher than that of the control group. Hepatopancreatic digestive enzyme activity is a key indicator of digestive capacity in crustaceans, reflecting physiological responses under different environmental or nutritional conditions [37]. These findings indicated that in August, crabs in the experimental group had enhanced capacities to absorb and utilize proteins, lipids, and carbohydrates compared to the control group, which is consistent with their improved growth performance. In October, female crabs in the experimental group showed higher protease activity and more efficient protein absorption and utilization than those in the control group, consistent with their superior growth performance.

### 4.3. Effects of Feeding Regimens on Hepatopancreas and Intestinal Health

In October, the alkaline phosphatase (AKP) and acid phosphatase (ACP) levels of male crabs in the experimental group were significantly higher than those in the control group. AKP [38] and ACP [39] constitute the first line of non-specific immune defense in Chinese mitten crabs and are key indicators of innate immune function [40]. This might indicate that the immune capacity of the male crabs in the experimental group is superior to that of the control group. Malondialdehyde (MDA), a lipid peroxidation byproduct of free radical reactions, reflects the degree of oxidative stress and cellular injury. Elevated MDA levels indicate more severe oxidative damage and are negatively correlated with hepatopancreatic antioxidant capacity [41].

Glutamic pyruvic transaminase (GPT) is a key transaminase primarily found in animal cells, where they play essential roles in protein metabolism. As a critical enzyme in amino acid catabolism, increased GPT activity enhances energy production from various amino acids [42]. The elevated transaminase activities in the hepatopancreas of the experimental group suggest enhanced amino acid metabolism and improved protein utilization, which likely contributed to increased protein deposition—findings consistent with the observed growth performance. During tissue necrosis, increased membrane permeability leads to the release of intracellular transaminases into the plasma [43]. Hemolymph transaminase activity serves as an indicator of hepatopancreatic damage severity. Although GPT levels in the hemolymph of the experimental group were significantly elevated, they remained below the physiological threshold (refer to the human body 40 U/L) for healthy crabs, which contrasts with the conclusions of Matilla B, who reported that alanyl-glutamine supplementation had no impact on hepatic metabolism [44]. It is also speculated that the observed elevation in GPT may be partly attributed to the crabs being in the reproductive phase [45]. In our future research, we may incorporate relevant hormone or reproductive stage indicators to confirm this hypothesis.

While some studies demonstrate the efficacy of stable, soluble glutamine dipeptides as amino acid supplements [46], others emphasize their rapid conversion to glutamine, which enhances immune and antioxidant responses in aquatic species [17,32]. The observed reversal in hepatopancreatic enzyme activities following prolonged dipeptide feeding, coupled with reduced MDA levels, suggests a time-dependent improvement in antioxidant defenses. The gender-specific response in GPT activity, without concurrent changes in other enzymes, may reflect sex-dependent metabolic adaptations. The differential hemolymph enzyme patterns, combined with their known subcellular localization [47,48] and histopathological observations, may indicate hepatopancreatic membrane integrity alterations. These findings collectively demonstrate the cross-species benefits of glutamine dipeptide supplementation for hepatopancreatic function, as evidenced in multiple aquatic species [18,19]. The sexually dimorphic enzyme responses, particularly in female crabs, highlight the need for gender-specific nutritional studies. The parallel intestinal health improvements further substantiate glutamine’s evolutionarily conserved gastrointestinal protective functions.

### 4.4. Effects of Feeding Regimens on Taste Quality of Edible Parts and Consumer Acceptance

#### 4.4.1. Taste Quality of Edible Parts

As the basic units of proteins, amino acids are crucial for organismal growth and development. Tissue free amino acid (FAA) levels correlate with nutritional value [49] and directly influence *E. sinensis* flavor perception [50]. ^15^N-labeled glutamine studies confirm amide nitrogen’s role in pyrazine synthesis [51]. This proves that the amide nitrogen in glutamine is conducive to the formation of flavor. Based on this, we speculate that the addition of glutamine dipeptide may also enhance the flavor of the Chinese mitten crab.

We detected 16 FAAs in the muscle/hepatopancreas and 15 in gonads. Glutamic acid and alanine dominated male gonads, while females showed arginine and alanine predominance. Serine and threonine were sexually dimorphic: undetectable in male and female gonads, respectively. Muscle FAA diversity (*n* = 16) was lower than Chen’s report (*n* = 21) but aligned with Yu’s findings (*n* = 17) [52,53].

Glutamic and aspartic acids constituted the core umami components in muscle [54,55]. Experimental males and females showed 85% and 55% higher muscle glutamic acid than controls, likely from glutamine hydrolysis. Alanine accumulation in male muscle and female gonads of the experimental group reflected dietary dipeptide utilization.

Hepatopancreatic FAA profiles differed significantly by sex, with alanine and arginine being predominant. PCA revealed that these variations critically influenced taste characteristics. Despite lower total FAAs in experimental females (*p* < 0.05), their elevated umami/sweet amino acid ratios (*p* < 0.05) suggested enhanced flavor potential.

Control females exhibited enhanced sourness (AHS) and sweetness (SCS) responses in PCA. E-tongue radar plots revealed high hepatopancreas sample similarity between groups, supported by PCA overlap. Muscle analyses showed that control females had stronger astringency (ANS), sweetness (SCS), and sourness (AHS) responses, correlating with their sweet amino acid content. Males displayed inverse but weaker trends.

Seasonal analysis (October) of edible tissues indicated experimental groups accumulated specific umami/sweet amino acids. Given sweet amino acids’ flavor impact [56] and bitter amino acids’ modulation effects, the e-tongue detected significant intergroup taste differences within sexes. The corresponding PCA showed minimal overlap, with female control muscle flavor advantage being modest (PC2 eigenvalue: 27.5%).

#### 4.4.2. Consumer Acceptance

Based on our analysis of the taste characteristics of Chinese mitten crabs in the previous text, we believe that to a certain extent, consumers’ acceptance of the experimental group’s Chinese mitten crabs will be higher, or it will not be lower than that of the control group’s Chinese mitten crabs. This is in line with a previous research conclusion, which stated that feeding with compound feed would increase consumers’ preference for the meat and roe of Chinese mitten crabs [57]. This is also consistent with the fact that the content of glutamic acid and aspartic acid, the core umami components in our muscles, has increased. The higher content of some free amino acids in the experimental group may endow it with higher nutritional value. We conducted an experiment similar to the taste panel verification. Most people considered that the taste of the Chinese mitten crabs in the experimental group was no less than that in the control group or even better than that in the control group. In conclusion, consumers’ acceptance of Chinese mitten crabs fed with compound feed throughout the process is acceptable.

## 5. Conclusions

Under the conditions of this experiment, Chinese mitten crab fed exclusively with glutamine dipeptide-supplemented formulated feed exhibited superior growth performance, enhanced hepatopancreatic antioxidant and digestive capacities, and improved intestinal health compared to those fed a mixture of frozen fish and formulated feed. In addition, the flavor characteristics of edible tissues are influenced by diet and gender. In the future, we will integrate gender-specific metabolic pathways to analyze the impact of gender. After crabs received dipeptide supplementation in their diet, the amino acid composition related to flavor was significantly improved. Of course, in this experiment focusing on the production frontline, limitations brought about by possible environmental changes due to the lack of repeat groups are objectively present, but they are also reasonable. However, these studies can still indicate to a certain extent that 5-month substitution of frozen fish with glutamine dipeptide-supplemented formulated feed represents a promising strategy for improving growth efficiency and edible quality in *E. sinensis* aquaculture. In the future, the potential long-term (throughout the entire crab-farming process) effects of supplementing glutamine dipeptide and consumers’ acceptance of feed-fed Chinese mitten crabs can be explored.

## Figures and Tables

**Table 1:** Growth performance of Chinese mitten crab in August under two feeding modes.

Indicator\Groups	CF	EF	CM	EM
Body Weight/g	86.38 ± 11.52	136.20 ± 10.52 *	123.84 ± 17.07	152.68 ± 11.97 *
Body Length/cm	5.40 ± 0.28	6.42 ± 0.16 *	5.89 ± 0.24	6.29 ± 0.19 *
Body Width/cm	5.77 ± 0.26	7.01 ± 0.12 *	6.38 ± 0.29	6.85 ± 0.23 *

Note: The data marked with * indicates a significant difference between the control group and the experimental group of the same sex (*p* < 0.05), while no * indicates no significant difference (*p* > 0.05). CF means control female; EF means experimental female; CM means control male; EM means experimental male.

**Table 2:** Growth performance and physical indexes of Chinese mitten crab in October under two feeding modes.

Indicator\Groups	CF	EF	CM	EM
Body Weight/g	122.93 ± 5.45	156.76 ± 9.68 *	154.05 ± 26.93	195.53 ± 6.45 *
Body Length/cm	5.90 ± 0.27	6.54 ± 0.21 *	6.14 ± 0.42	6.45 ± 0.21
Body Width/cm	6.42 ± 0.10	7.04 ± 0.35 *	6.69 ± 0.38	7.11 ± 0.30
HSI/%	7.76 ± 0.74	7.57 ± 1.26	7.55 ± 1.61	6.69 ± 0.35
GSI/%	8.41 ± 1.28	7.54 ± 2.86	1.85 ± 0.51	2.23 ± 0.61
MY/%	20.29 ± 1.05	18.80 ± 3.25	18.89 ± 3.86	22.70 ± 1.38
TEY/%	35.46 ± 2.47	35.53 ± 2.35	29.51 ± 3.50	31.62 ± 0.55

Note: The data marked with * indicates a significant difference between the control group and the experimental group of the same sex (*p* < 0.05), while no * indicates no significant difference (*p* > 0.05).

**Table 3:** Hepatopancreas enzyme activity and lipid oxidation production of Chinese mitten crab under two feeding modes in August and October.

Month	Groups	AKP Kunitz Units/gprot	ACP Kunitz Units/gprot	MDA nmol/mgprot	GPT U/gprot	GOT U/gprot
August	CF	34.74 ± 4.42	8.13 ± 0.92	0.83 ± 0.09	1.95 ± 0.45	3.06 ± 1.87
	EF	53.07 ± 2.86 *	7.82 ± 0.87	1.68 ± 0.05 *	1.97 ± 0.48	9.57 ± 4.33
	CM	52.55 ± 7.41	6.56 ± 0.50	1.83 ± 0.15 *	1.72 ± 0.10	3.13 ± 0.00
	EM	75.88 ± 5.46 *	10.76 ± 1.53 *	1.01 ± 0.10	9.84 ± 0.35 *	11.90 ± 6.25
October	CF	146.35 ± 4.78 *	14.65 ± 1.64	5.61 ± 0.52 *	6.28 ± 1.12	10.39 ± 3.46
	EF	130.54 ± 6.11	17.56 ± 1.55	4.29 ± 0.19	19.89 ± 1.82 *	9.07 ± 4.71
	CM	48.46 ± 1.88	5.68 ± 0.76	13.92 ± 0.40 *	5.54 ± 1.22	11.05 ± 3.33
	EM	89.20 ± 2.80 *	11.38 ± 3.20 *	6.77 ± 0.24	4.96 ± 0.83	11.35 ± 4.04

Notes: The data marked with * indicates a significant difference between the control group and the experimental group of the same sex (*p* < 0.05), while no * indicates no significant difference (*p* > 0.05).

**Table 4:** Hemolymph enzyme activity and lipid oxidation production of Chinese mitten crab in August and October under two feeding modes.

Month	Groups	AKP (Kunitz Units/100 mL)	ACP (Kunitz Units/100 mL)	MDA (nmol/mL)	GPT (U/L)
August	CF	0.50 ± 0.34	4.01 ± 1.41	3.10 ± 0.27	10.91 ± 1.83 *
	EF	1.24 ± 0.14 *	5.36 ± 0.62	5.45 ± 0.14 *	4.39 ± 0.18
	CM	0.32 ± 0.11	3.09 ± 0.46	6.16 ± 1.11	4.38 ± 1.30
	EM	0.33 ± 0.09	4.22 ± 0.81	6.47 ± 2.67	18.00 ± 4.96 *
October	CF	0.69 ± 0.14 *	2.90 ± 0.08 *	4.04 ± 0.27	4.41 ± 0.88
	EF	0.36 ± 0.12	1.21 ± 0.32	3.33 ± 0.59	15.58 ± 10.14 *
	CM	0.15 ± 0.13	1.82 ± 0.39	3.88 ± 0.24	1.90 ± 0.51
	EM	0.57 ± 0.11 *	3.30 ± 0.49 *	3.88 ± 0.41	4.92 ± 1.01 *

Notes: The data marked with * indicates a significant difference between the control group and the experimental group of the same sex (*p* < 0.05), while no * indicates no significant difference (*p* > 0.05).

**Table 5:** Hepatopancreas digestive enzyme activity of Chinese mitten crab under two feeding modes in August and October.

Month	Groups	Protease (U/mgprot)	Amylase (U/gprot)	Lipase (U/mgprot)
August	CF	633.97 ± 290.29	6.57 ± 1.41	8.11 ± 1.25
	EF	4397.76 ± 472.58 *	7.18 ± 2.16	36.74 ± 0.86 *
	CM	107.64 ± 53.80	8.85 ± 1.76	14.83 ± 0.69
	EM	1880.24 ± 130.35 *	10.40 ± 1.38	20.48 ± 2.15 *
October	CF	2061.46 ± 55.22	15.36 ± 2.05 *	19.83 ± 0.79 *
	EF	3536.67 ± 61.19 *	8.09 ± 1.95	6.27 ± 0.78
	CM	2535.18 ± 71.53 *	2.65 ± 0.83	25.56 ± 2.43
	EM	997.71 ± 2.44	20.84 ± 3.29 *	28.53 ± 5.86

Notes: The data marked with * indicates a significant difference between the control group and the experimental group of the same sex (*p* < 0.05), while no * indicates no significant difference (*p* > 0.05).

**Table 6:** Muscle routine (wet weight) of Chinese mitten crab under two feeding modes in October.

Indicator\Groups	CF	EF	CM	EM
Moisture/%	76.01 ± 0.73	82.42 ± 0.77 *	77.19 ± 0.39	76.79 ± 0.56
Ash/%	1.67 ± 0.12	1.28 ± 0.03	1.63 ± 0.06	1.71 ± 0.11
Crude protein/%	19.79 ± 0.12	14.57 ± 0.11	18.05 ± 0.04	18.53 ± 0.33
Crude fat/%	1.15 ± 0.09	0.84 ± 0.12	0.97 ± 0.10	0.84 ± 0.32

Notes: The data marked with * indicates a significant difference between the control group and the experimental group of the same sex (*p* < 0.05), while no * indicates no significant difference (*p* > 0.05).

**Table 7:** Content of free amino acids in muscle (dry weight) of Chinese mitten crab under two feeding modes in October.

AA(mg/g)	Taste	CF	EF	CM	EM
Asp	umami (+)	1.28 ± 0.27	1.27 ± 0.07	1.95 ± 0.04 *	1.46 ± 0.28
Glu	umami (+)	1.87 ± 1.12	2.88 ± 0.14	1.40 ± 0.79	2.58 ± 1.62
Ser	sweet (+)	3.00 ± 0.78	3.15 ± 0.13	1.81 ± 0.79	5.02 ± 0.90 *
His	sweet (+)	1.49 ± 0.05	1.41 ± 0.05	1.15 ± 0.02	1.53 ± 0.04 *
Gly	sweet (+)	20.90 ± 0.58	20.11 ± 0.36	19.87 ± 0.10	22.30 ± 0.49 *
Thr	sweet (+)	1.34 ± 0.20	2.73 ± 0.12 *	1.16 ± 0.59	2.60 ± 0.19 *
Ala	sweet (+)	18.45 ± 0.53	17.64 ± 0.39	15.31 ± 0.04	20.24 ± 0.54 *
Arg	sweet (+)	29.97 ± 1.23	28.58 ± 1.25	29.26 ± 0.40	28.01 ± 0.91
Tyr	bitter (−)	1.15 ± 0.04 *	1.04 ± 0.04	0.79 ± 0.01	0.96 ± 0.02 *
Val	bitter (−)	1.27 ± 0.05	1.19 ± 0.04	1.10 ± 0.01	1.44 ± 0.04 *
Met	bitter (−)	1.35 ± 0.05 *	1.24 ± 0.04	1.34 ± 0.01 *	1.18 ± 0.04
Phe	bitter (−)	1.76 ± 0.05	1.71 ± 0.02	1.40 ± 0.02	1.73 ± 0.01 *
Ile	bitter (−)	0.65 ± 0.02	0.60 ± 0.02	0.40 ± 0.00	0.52 ± 0.01 *
Leu	bitter (−)	1.45 ± 0.04 *	1.35 ± 0.04	1.13 ± 0.01	1.47 ± 0.04 *
Lys	bitter (−)	1.82 ± 0.08 *	1.64 ± 0.05	1.75 ± 0.02	2.60 ± 0.07 *
Pro	bitter (−)	14.5 ± 0.45	14.07 ± 0.32	16.29 ± 0.19 *	15.53 ± 0.35
Umami FAA		3.16 ± 1.40	4.15 ± 0.21	3.35 ± 0.75	4.05 ± 1.90
Sweet FAA		48.42 ± 1.76	46.22 ± 1.64	68.56 ± 1.94	79.71 ± 3.06 *
Bitter FAA		50.67 ± 1.99	50.23 ± 1.22	24.21 ± 0.27	25.43 ± 0.58 *
TAA		102.25 ± 5.15	100.6 ± 3.07	96.11 ± 1.46	109.18 ± 5.54 *
Umami FAA/TAA (%)		3.05 ± 1.21	4.12 ± 0.08	3.50 ± 0.84	3.65 ± 1.56
Sweet FAA/TAA (%)		47.38 ± 0.66 *	45.94 ± 0.23	71.32 ± 0.94	73.04 ± 0.91

Note: +, positive taste; −, negative taste. The following tables are the same. The data marked with * indicates a significant difference between the control group and the experimental group of the same sex (*p*<0.05), while no * indicates no significant difference (*p* > 0.05).

**Table 8:** Content of free amino acids in the hepatopancreas of Chinese mitten crab under two feeding modes in October.

AA (mg/g)	Taste	CF	EF	CM	EM
Asp	umami (+)	0.59 ± 0.03 *	0.06 ± 0.00	0.30 ± 0.00	0.29 ± 0.05
Glu	umami (+)	1.18 ± 0.03 *	0.67 ± 0.00	1.71 ± 0.02	1.73 ± 0.19
Ser	sweet (+)	1.21 ± 0.04 *	0.60 ± 0.02	1.48 ± 0.01	1.28 ± 0.15
His	sweet (+)	0.48 ± 0.00 *	0.34 ± 0.01	0.59 ± 0.03	0.62 ± 0.08
Gly	sweet (+)	0.82 ± 0.02 *	0.73 ± 0.00	1.16 ± 0.05	1.14 ± 0.09
Thr	sweet (+)	0.95 ± 0.02 *	0.42 ± 0.01	1.33 ± 0.02	1.25 ± 0.20
Ala	sweet (+)	2.19 ± 0.07	2.14 ± 0.06	3.30 ± 0.04	3.39 ± 0.11
Arg	sweet (+)	2.89 ± 0.08 *	2.06 ± 0.03	3.11 ± 0.02	3.26 ± 0.34
Tyr	bitter (−)	0.75 ± 0.07 *	0.40 ± 0.02	0.88 ± 0.02	0.93 ± 0.11
Val	bitter (−)	1.19 ± 0.00 *	0.75 ± 0.02	1.44 ± 0.03	1.43 ± 0.15
Met	bitter (−)	0.08 ± 0.01 *	0.23 ± 0.02	0.48 ± 0.03	0.39 ± 0.11
Phe	bitter (−)	1.73 ± 0.05 *	1.29 ± 0.02	2.30 ± 0.02	2.21 ± 0.21
Ile	bitter (−)	0.70 ± 0.01 *	0.40 ± 0.01	0.91 ± 0.01	0.92 ± 0.13
Leu	bitter (−)	1.48 ± 0.02 *	0.87 ± 0.02	2.09 ± 0.05	2.08 ± 0.31
Lys	bitter (−)	1.98 ± 0.04 *	0.77 ± 0.04	2.32 ± 0.01	2.24 ± 0.37
Pro	bitter (−)	0.75 ± 0.01 *	0.22 ± 0.01	1.19 ± 0.02	1.20 ± 0.15
Umami FAA		1.77 ± 0.05 *	0.73 ± 0.00	2.01 ± 0.02	2.03 ± 0.24
Sweet FAA		5.07 ± 0.14 *	4.20 ± 0.09	6.42 ± 0.02	6.64 ± 0.45
Bitter FAA		11.78 ± 0.07 *	7.01 ± 0.10	16.17 ± 0.04	15.75 ± 2.08
TAA		18.63 ± 0.27 *	11.94 ± 0.19	24.60 ± 0.00	24.42 ± 2.77
Umami FAA/TAA (%)		9.52 ± 0.15	17.37 ± 0.37 *	8.17 ± 0.09	8.29 ± 0.06
Sweet FAA/TAA (%)		27.24 ± 0.38	35.16 ± 0.18 *	26.09 ± 0.00	27.30 ± 1.26

Notes: The following tables are the same. The data marked with * indicates a significant difference between the control group and the experimental group of the same sex (*p* < 0.05), while no * indicates no significant difference (*p* > 0.05).

**Table 9:** Content of free amino acids in gonads of Chinese mitten crab under two feeding modes in October.

AA (mg/g)	Taste	CF	EF	CM	EM
Asp	umami (+)	0.07 ± 0.00	0.07 ± 0.06	1.77 ± 0.18	1.64 ± 0.04
Glu	umami (+)	0.68 ± 0.07	0.60 ± 0.00	5.52 ± 1.76	6.95 ± 0.28
Ser	sweet (+)	0.24 ± 0.09	0.12 ± 0.00	0.00	0.00
His	sweet (+)	0.42 ± 0.01	0.41 ± 0.00	0.50 ± 0.02	0.55 ± 0.02
Gly	sweet (+)	0.55 ± 0.04	0.61 ± 0.00	1.17 ± 0.04	1.37 ± 0.01 *
Thr	sweet (+)	0.00	0.00	4.02 ± 0.13	3.97 ± 0.15
Ala	sweet (+)	1.39 ± 0.06	1.56 ± 0.01 *	6.33 ± 0.16	6.66 ± 0.19
Arg	sweet (+)	4.88 ± 0.18 *	4.30 ± 0.03	1.78 ± 0.06	1.85 ± 0.09
Tyr	bitter (−)	0.31 ± 0.01	0.40 ± 0.02 *	0.45 ± 0.01	1.69 ± 0.05 *
Val	bitter (−)	0.32 ± 0.01 *	0.28 ± 0.00	0.54 ± 0.01	0.62 ± 0.01 *
Met	bitter (−)	0.28 ± 0.01 *	0.23 ± 0.00	0.33 ± 0.01 *	0.22 ± 0.01
Phe	bitter (−)	1.01 ± 0.02 *	0.95 ± 0.01	1.15 ± 0.03	1.26 ± 0.01 *
Ile	bitter (−)	0.12 ± 0.00 *	0.10 ± 0.00	0.51 ± 0.02	0.63 ± 0.02 *
Leu	bitter (−)	0.23 ± 0.01 *	0.21 ± 0.00	0.63 ± 0.02	0.86 ± 0.03 *
Lys	bitter (−)	1.31 ± 0.04 *	0.82 ± 0.01	1.06 ± 0.03	1.30 ± 0.05 *
Pro	bitter (−)	0.49 ± 0.02	0.28 ± 0.28	4.20 ± 0.28	4.57 ± 0.13
Umami FAA		0.75 ± 0.06	0.67 ± 0.05	7.29 ± 1.94	8.59 ± 0.32
Sweet FAA		7.47 ± 0.19 *	7.00 ± 0.05	13.80 ± 0.41	14.39 ± 0.46
Bitter FAA		4.10 ± 0.12 *	3.30 ± 0.24	7.24 ± 0.32	9.65 ± 0.27 *
TAA		12.31 ± 0.24 *	10.98 ± 0.34	28.33 ± 2.03	32.62 ± 1.05 *
Umami FAA/TAA(%)		6.07 ± 0.63	6.13 ± 0.31	25.51 ± 5.02	26.32 ± 0.13
Sweet FAA/TAA(%)		60.68 ± 0.34	63.81 ± 1.55 *	48.8 ± 2.05 *	44.11 ± 0.00

Notes: The following tables are the same. The data marked with * indicates a significant difference between the control group and the experimental group of the same sex (*p* < 0.05), while no * indicates no significant difference (*p* > 0.05).

## Data Availability

Data are contained within the article.
